# Spatiotemporal Variations of Indoor PM_2.5_ Concentrations in Nanjing, China

**DOI:** 10.3390/ijerph16010144

**Published:** 2019-01-07

**Authors:** Zhijuan Shao, Xiangjun Yin, Jun Bi, Zongwei Ma, Jinnan Wang

**Affiliations:** 1 State Key Laboratory of Pollution Control & Resource Reuse, School of the Environment, Nanjing University, Nanjing 210023, China; shaozhijuan@126.com (Z.S.); jbi@nju.edu.cn (J.B.); wangjn@caep.org.cn (J.W.); 2 Nanjing Urban Planning & Research Center, Nanjing 210029, China; yinxiangjun0602@163.com; 3 Jiangsu Collaborative Innovation Center of Atmospheric Environment and Equipment Technology, Nanjing University of Information Science & Technology, Nanjing 210044, China; 4 State Environmental Protection Key Laboratory of Environmental Planning and Policy Simulation, Chinese Academy for Environmental Planning, Beijing 100012, China

**Keywords:** indoor PM_2.5_, indoor/outdoor ratio, CONTAM, health impact

## Abstract

Indoor fine particulate matter (PM_2.5_) is important since people spend most of their time indoors. However, knowledge of the spatiotemporal variations of indoor PM_2.5_ concentrations within a city is limited. In this study, the spatiotemporal distributions of indoor PM_2.5_ levels in Nanjing, China were modeled by the multizone airflow and contaminant transport program (CONTAM), based on the geographically distributed residences, human activities, and outdoor PM_2.5_ concentrations. The accuracy of the CONTAM model was verified, with a good agreement between the model simulations and measurements (r = 0.940, *N* = 110). Two different scenarios were considered to examine the building performance and influence of occupant behaviors. Higher PM_2.5_ concentrations were observed under the scenario when indoor activities were considered. Seasonal variability was observed in indoor PM_2.5_ levels, with the highest concentrations occurring in the winter and the lowest occurring in the summer. Building characteristics have a significant effect on the spatial distribution of indoor PM_2.5_ concentrations, with multistory residences being more vulnerable to outdoor PM_2.5_ infiltration than high-rise residences. The overall population exposure to PM_2.5_ in Nanjing was estimated. It would be overestimated by 16.67% if indoor exposure was not taken into account, which would lead to a bias in the health impacts assessment.

## 1. Introduction

Due to the rapid urbanization and economic growth of China over the past few decades, the nation has experienced extremely high levels of air pollution, with PM_2.5_ as the dominant pollutant [1,2]. PM_2.5_ was identified as one of the leading risk factors for the global burden of disease (GBD) [3]. Epidemiological studies have demonstrated the associations between PM_2.5_ exposure and a range of health effects, such as cardiovascular and respiratory diseases, cancer, and preterm birth [4,5,6,7]. Although people spend more than 80% of their time indoors [8,9,10], most air pollution health studies typically use a few fixed-site measurements of PM_2.5_ to represent human exposure concentrations [11,12,13], due to the lack of indoor PM_2.5_ concentration data. The difference between indoor and outdoor PM_2.5_ concentrations may bias health effect assessments in epidemiological studies [14,15,16].

A common approach for studying indoor PM_2.5_ concentrations is field measurements, and a number of studies have monitored PM_2.5_ in residences [17,18,19]. However, indoor environments are complicated since indoor PM_2.5_ levels are affected by the infiltration of outdoor PM_2.5_ concentrations, emissions from indoor sources, and the removal of internal air via deposition, filtration, and exfiltration [20]. Due to the high cost and labor demand of indoor sampling, field measurement studies usually include data only from a few residences in a specific period [21,22], and the research on indoor air pollution on a long time scale and a large spatial scale is lacking. This will make it difficult to understand the overall level of indoor air pollution in a regional area or a city. Modeling methods have been developed to overcome the limitations of indoor sampling. CONTAM (contaminant transport program) is a multizone indoor air quality and ventilation analysis program, which is designed to help determine airflows, contaminant concentrations, and personal exposure in buildings by the National Institute of Standards and Technology, USA [23]. The program has been widely used to assess the indoor air quality performance of multiple buildings. Shrubsole et al. using CONTAM modeled the change in indoor PM_2.5_ exposure concentrations in London’s domestic stock due to the energy-efficient refurbishment [24]. Fabian et al. simulated the indoor concentrations of PM_2.5_ in multifamily housing in Boston via the CONTAM program [25]. However, the application of the CONTAM model for indoor PM_2.5_ simulations is rare in China. Few studies focused on indoor gaseous pollutant transport [26] and air infiltration rate distributions [27]. The characteristics of buildings and the meteorological features in China are different from those in other countries. As such, whether or not the model is appropriate for modeling PM_2.5_ concentrations in Chinese residences still needs to be investigated.

Moreover, most of the indoor PM_2.5_ studies mainly focused on the indoor levels of PM_2.5_, source apportionment, and the indoor-outdoor relationship [17,18,19,28,29,30]. There has been little research on the spatial and temporal distributions of indoor PM_2.5_ concentrations. Few studies had examined the spatial and temporal variation of PM_2.5_ concentrations within a single house or building [31,32,33]. However, the spatiotemporal distribution of indoor PM_2.5_ on the urban scale was seldom studied. Other researchers had found the large difference of indoor PM concentrations between cities and explored the associations between indoor PM exposure and health effects [21,34,35], but the variations of building characteristics and their influence on indoor PM concentrations were rarely considered in these studies. Building characteristics (e.g., building type, age, and level) have been reported to have an influence on indoor air pollution [36,37,38]. The distribution of the building envelope can modify the distribution of population exposure to PM_2.5_ from the outdoors across an urban area [39]. A study on the spatiotemporal variations of indoor PM_2.5_ concentrations is important for understanding the overall level of PM_2.5_ in residences in a city and improving the accuracy of exposure assessment of PM_2.5._

Therefore, in this study, we performed a spatiotemporal analysis of PM_2.5_ in residences in Nanjing. The primary purpose of this study was to: (1) investigate the ability of the CONTAM model to predict PM_2.5_ concentrations in multiple residences in China by comparing the model simulations with the experimental measurements, (2) model the spatiotemporal distributions of PM_2.5_ concentrations in residences across Nanjing via CONTAM based on the geographically distributed residential buildings, and (3) estimate the overall population exposure to PM_2.5_ in Nanjing and the associated health consequences by taking into account both indoor and outdoor PM_2.5_ concentrations.

## 2. Materials and Methods

### 2.1. Study Area

**Figure 1:**
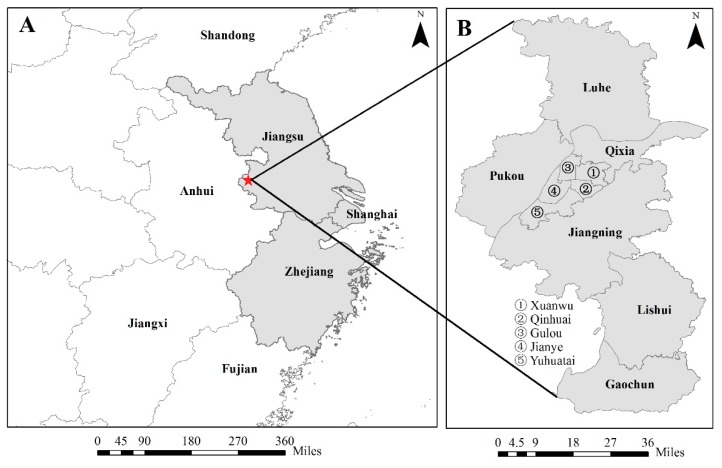
The location of the study area. (**A**) The Yangtze River Delta. (**B**) Nanjing.

The study was carried out in Nanjing, which is a major city in the Yangtze River Delta in East China (31°14’–32°37’ N, 118°22’–119°14’ E) (Figure 1). The city is divided into eleven districts including Xuanwu, Qinhuai, Jianye, Gulou, Pukou, Qixia, Yuhuatai, Jiangning, Luhe, Lishui, and Gaochun, with a population of more than 8.2 million people [40]. The air pollution in Nanjing is serious, with an average annual PM_2.5_ concentration of 47.90 μg/m^3^ in 2016 [41], which is much higher than the annual mean standard (35 μg/m^3^) of National Ambient Air Quality Standards suggested by the Ministry of Environmental Protection of China (MEPC). Nanjing has a subtropical and humid climate, with a cold winter and hot summer. In the spring and the autumn, the temperature is appropriate. Residential buildings are mainly naturally ventilated, and air conditionings are often used for heating and cooling by occupants in the winter and the summer. Natural gas is usually used as the fuel for cooking in families in Nanjing.

### 2.2. Study Design

**Figure 2:**
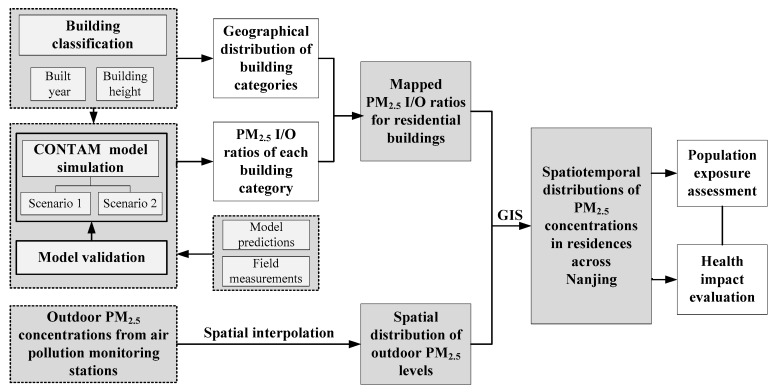
A flow chart of the study for indoor PM_2.5_ concentrations in residences. (I/O: indoor/outdoor).

A spatiotemporal analysis of PM_2.5_ concentrations in residences across the city was performed in this study. The project workflow can be seen in Figure 2.

First, we categorized the residential buildings in Nanjing and mapped the geographically distribution of the building categories. Second, the indoor/outdoor (I/O) ratios were modeled using the CONTAM model for each building category. The accuracy of the model for indoor PM_2.5_ prediction was validated by the field measurements. Third, the seasonal and annual I/O ratios of PM_2.5_ for each building type were calculated and assigned to the mapped building categories. The spatial distribution of outdoor PM_2.5_ levels in four seasons was calculated via spatial interpolation using data from air pollution monitoring stations. The outdoor PM_2.5_ concentrations were then matched with the spatially distributed I/O ratios and the spatiotemporal indoor PM_2.5_ concentrations across the city were calculated. Lastly, the population exposure concentrations to PM_2.5_ in Nanjing and their health impacts were estimated based on both indoor and outdoor PM_2.5_ exposures.

### 2.3. Residential Building Category

The residential buildings in Nanjing were used for the study of spatial and temporal distribution of indoor PM_2.5_. In total, about 209,764 residential buildings were involved in this study. The mapped residential building data were obtained from the Nanjing Urban Planning & Research Center and included the geographic locations of building footprints, names, and building stories. The age information for each building was supplemented by data from a real estate website (http://nj.house365.com/).

Building height is an important factor that can influence air permeability performance, and the Chinese government has set a related design standard for multistory and high-rise residences [42,43]. Therefore, the residences in Nanjing can be roughly divided into two categories: multistory residential buildings (≤7 stories) and high-rise residential buildings (>7 stories). In each category, the buildings were further classified into four groups according to the construction year (before 1990, 1991–2000, 2001–2010, and after 2011). Building orientation is another influencing factor of the building infiltration rate [44]. According to our previous survey [17], the residential buildings were mainly north/south facing. Therefore, the orientation of residential buildings in this study was considered to be north to south. High-rise buildings built before 1990 in Nanjing were rare [17]. Therefore, the residential buildings were divided into 8 groups, as shown in Table 1.

### 2.4. PM_2.5_ I/O Ratios of Residential Buildings

#### 2.4.1. CONTAM Model Simulation

**Figure 3:**
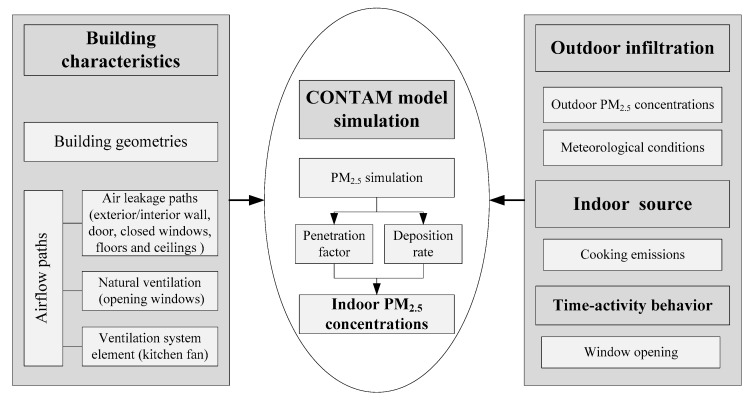
Conceptual diagram of the CONTAM model simulation approach.

The CONTAM model was used to model the hourly I/O ratios of each residential building in a whole year [23]. Figure 3 illustrates an outline for the modeling approach. The specific layout of each building and its airflow paths was set up in the sketchpad of CONTAM (Figure S1). Simulations were run to investigate the influence of building characteristics and human activities on indoor PM_2.5_ concentrations. The input parameters of building characteristics in the model are presented in Table 1 and Table S4. Other detailed information about the model simulation are presented in the Supporting Information, in the section “CONTAM building model”.

Indoor PM_2.5_ simulation for each single building was run for the whole year of 2016 using the Test Reference Year-type hourly weather file for Nanjing, China (https://energyplus.net/weather). Outdoor hourly PM_2.5_ concentrations from nine monitoring stations in Nanjing were obtained from the China Environmental Monitoring Center (CEMC). The average hourly concentrations of these nine stations were calculated and used for modeling.

Cooking was considered as the indoor source for PM_2.5_ in this study. Cooking emission was modeled three times a day in each building, according to the study conducted by Fabian et al. [25]. The detailed information for the emission rate and deposition rate of PM_2.5_ are shown in Table S1 [46,47].

Residential buildings in China normally do not use mechanical ventilation systems, and all the residential buildings were considered naturally ventilated. Only kitchen exhaust fans during cooking were set up in the model, with the airflow of 15 m^3^/min.

The indoor temperatures in the summer and winter were set at 26 °C and 12 °C, respectively, according to the thermo environmental design standard for residential buildings [48]. In the spring and autumn, the temperature is suitable in Nanjing and people often keep windows opened for ventilation. Thus, the indoor residential temperature in the two seasons were set at 20 °C, which is similar to the average outdoor temperature in the spring and autumn [40].

#### 2.4.2. Scenario Analysis

Two different scenarios were simulated to examine the residential building performance and influence of occupant behavior.

Scenario 1: Only the infiltration of outdoor air was modeled via the building components due to the permeability of the externally exposed facades of the buildings (e.g., exterior walls, windows, and doors).

Scenario 2: Windows were opened by occupants, and cooking emissions were considered. The ventilation time and schedule for opening windows was set according to the Exposure Factors Handbook of Chinese Population (Adults) (Table S2) [10]. The cooking emission was modeled three times a day, according to the study conducted by Burke et al. [46].

#### 2.4.3. Data Collection and Analysis

The all-year hourly indoor PM_2.5_ concentrations in each residential building under the two scenarios were output from the CONTAM models. The hourly I/O ratios were calculated for each building category, according to the outdoor PM_2.5_ concentrations. Then, the seasonal and annual mean I/O ratios were calculated. For the building R08, the construction year was unknown. The I/O ratio could not be estimated due to the lack of the effective leakage area of the exterior wall. To deal with this problem, the mean I/O ratio of the multistory buildings (R01 to R04) was calculated and assigned to building R08. The I/O ratios for each type of residential building were then summarized according to the two scenarios. We assumed that the hourly, seasonal, and annual I/O ratios of PM_2.5_ for each building were constant and did not change with the outdoor PM_2.5_ concentrations. The results of the I/O ratios were then used to estimate PM_2.5_ concentrations in residences across the city.

#### 2.4.4. Validation of the CONTAM Model

The ability of the CONTAM model for indoor PM_2.5_ prediction was verified by comparing the modeled concentrations with the measurements. Indoor/outdoor PM_2.5_ concentrations in 110 families in Nanjing were measured in 2016, and the characteristics of each family were investigated. Details of the measurements and data analysis were described in our previous study [17]. In this study, the specific layout of each residence was set up in the CONTAM sketchpad, according to its building characteristics. The input data of the models was set as described in Section 2.4.1. In addition to the cooking emissions, indoor smoking was also considered because there were smokers in some of the sampled families. An emission rate of 0.99 mg/min [24], with a deposition rate of 0.10 /h [49], was used for the smoking source. The schedules of window opening and indoor source emissions in each family were obtained from the time-activity survey during sampling. Indoor air simulations for the model validation were run for 24 h, according to the outdoor PM_2.5_ concentrations obtained from field measurements.

The modeled and measured 24-h average indoor PM_2.5_ concentrations in each family were compared using the American Society for Testing and Materials (ASTM) D5157 standard guide for the statistical evaluation of indoor air quality models [50,51]. Three statistical parameters (correlation coefficient (r), regression slope (M), and regression intercept (b)) were used to evaluate the accuracy of the indoor PM_2.5_ concentration predictions. Furthermore, three additional parameters, including the normalized mean square error (NMSE), fractional bias (FB), and fractional bias of variance (FS), were calculated.

### 2.5. Spatiotemporal Distributions of Indoor PM_2.5_ Concentrations

#### 2.5.1. Outdoor PM_2.5_ Concentrations

Hourly outdoor PM_2.5_ concentrations in 2016 were obtained from the CEMC measurements. Data from 221 air pollution monitoring stations, which were in the area of 6°×6° including Nanjing, were selected. The seasonal and annual mean PM_2.5_ concentrations at each station were calculated. Kriging interpolation was used to create the spatially resolved (100 m × 100 m) outdoor PM_2.5_ concentrations. Then, the data for Nanjing were extracted.

#### 2.5.2. Mapped PM_2.5_ I/O Ratios for Residential Buildings

The mapped footprints of residential buildings were obtained from the Nanjing Urban Planning & Research Center. The information of the building classification was added into each building, according to the building story and construction year in the geographic information system (GIS). Then, the CONTAM modeled seasonal and annual I/O ratios for each building were added into the database based on the building classifications. Lastly, the spatial distribution of PM_2.5_ I/O ratios for residential buildings in Nanjing was mapped (100 m×100 m). The mean seasonal and annual I/O ratios for each grid were calculated, and the results were mapped to show the differences in I/O ratios for residences in each grid across Nanjing.

#### 2.5.3. GIS Integration

Outdoor PM_2.5_ concentration data processed in 2.5.1 and the I/O ratio data mentioned in Section 2.5.2 were integrated by GIS through a spatial join. Indoor PM_2.5_ concentrations were estimated by multiplying the outdoor PM_2.5_ concentrations with corresponding I/O ratios. Both the seasonal and annual indoor PM_2.5_ concentrations for scenarios 1 and 2 were estimated. The spatial and temporal distributions of the indoor PM_2.5_ concentrations were mapped across the city.

### 2.6. Exposure Estimation and Health Impact Evaluation

The annual PM_2.5_ exposure was calculated based on the indoor and outdoor concentrations and the average time spent indoors and outdoors, as seen in Equation (1).

(1) $$C=C_{in}\times T_{in}+C_{out}\times T_{out}$$

where C is the annual PM_2.5_ exposure concentration, $C_{in}$ is the annual PM_2.5_ concentrations in residences estimated in this study, $C_{out}$ is the annual outdoor PM_2.5_ concentration, and $T_{in}$ and $T_{out}$ is the average daily time that residents spend indoors and outdoors, which is obtained from the Exposure Factors Handbook of Chinese Population (adults) [10].

Integrated exposure-response model was adopted to estimate the PM_2.5_ exposure-induced relative risks (RRs) of lung cancer (LC), cerebrovascular disease (stroke), ischemic heart disease (IHD), and chronic obstructive pulmonary disease (COPD) among the population in Nanjing [52]. The relative risk (RR) was calculated by Equation (2).

(2) $$\mathrm{RRC}=\{\begin{array}{cc} 1+\propto \left(1-e^{-\gamma {\left(c-c_{0}\right)}^{\delta }}\right), & if\;C>C_{0}\\ 1\;\;\;\;\;\;\;\;\;\;\;\;\;\;\;\;\;\;\;\;\;\;\;\;\;\;\;\;\;\;\;\;\;\;\;\;, & else \end{array}$$

where C is the annual PM_2.5_ exposure concentration, C_0_ is the counterfactual concentration, and α, γ, and δ are parameters used to describe the different shapes of the C-R curve among various diseases (Table S3) [53].

Then, the health impact of PM_2.5_ was calculated using the equation suggested by Ostro [54], as shown in Equation (3).

(3) $$\mathrm{ED}=\left(1-\frac{1}{RR}\right)\times B\times P$$

where ED is the excess death caused by PM_2.5_ for LC, stroke, IHD and COPD. B is the incidence of a given health impact for all ages and both genders obtained from GBD [55]. P is the population of Nanjing obtained from the Nanjing Statistical Yearbook [40].

### 2.7. Sensitivity Analysis

To explore the sensitivity of the CONTAM model to variations in the input parameters, a sensitivity analysis was carried out in this study. Five parameters including the leakage area of the exterior wall, exterior doors/windows, the penetration factor, the deposition rate, and the cooking emission rate were selected for analysis. The details of the method and results of sensitivity analysis are discussed in the Supporting Information in the section “Sensitivity Analysis” and Table S5–S6.

## 3. Results and Discussion

### 3.1. CONTAM Model Validation Result

**Figure 4:**
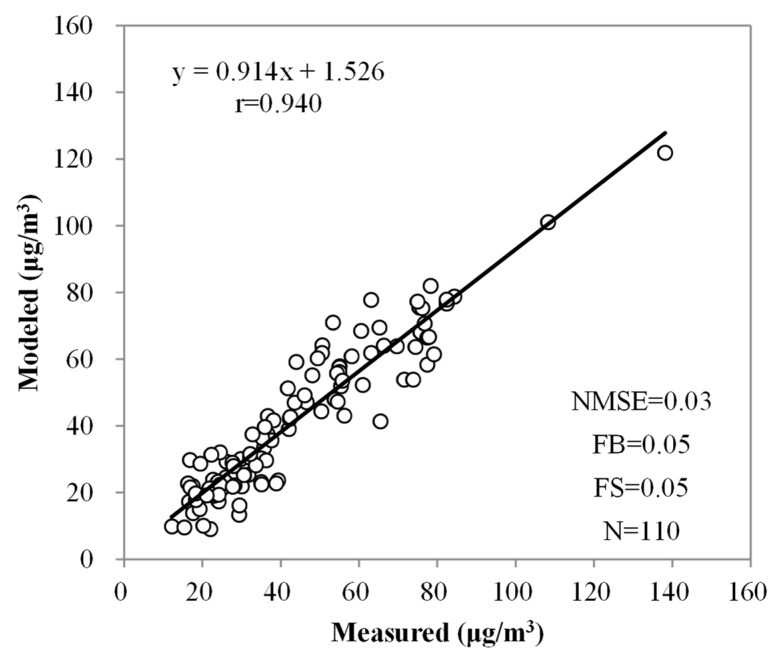
Scatterplots of the CONTAM model predictions versus measurements for 24-h average indoor PM_2.5_ concentrations. (NMSE: normalized mean square error, FB: fractional bias, and FS: fractional bias of variance)

The result of the CONTAM model validation is presented in Figure 4. A good agreement was found between the model simulations and the measurements (r = 0.940). Other parameters of the statistical analysis, including M (0.914), NMSE (0.03), FB (0.05), and FS (0.05), also showed that the agreement between the observations and model predictions is within an acceptable range [50]. The I/O ratios calculated from the measured and modeled indoor PM_2.5_ concentrations were also compared. The average modeled I/O ratio of the residences (1.07 ± 0.54) was close to the measured value (1.14 ± 0.56). The results in this study are consistent with previous studies, which showed that the simulated concentrations of particles in residences are in good agreement with the experimental data [25,50]. The results of the model validation demonstrated the model’s ability to predict PM_2.5_ concentrations in multiple residences in China with different building characteristics and indoor activities.

### 3.2. The I/O Ratios of Each Residential Building

**Figure 5:**
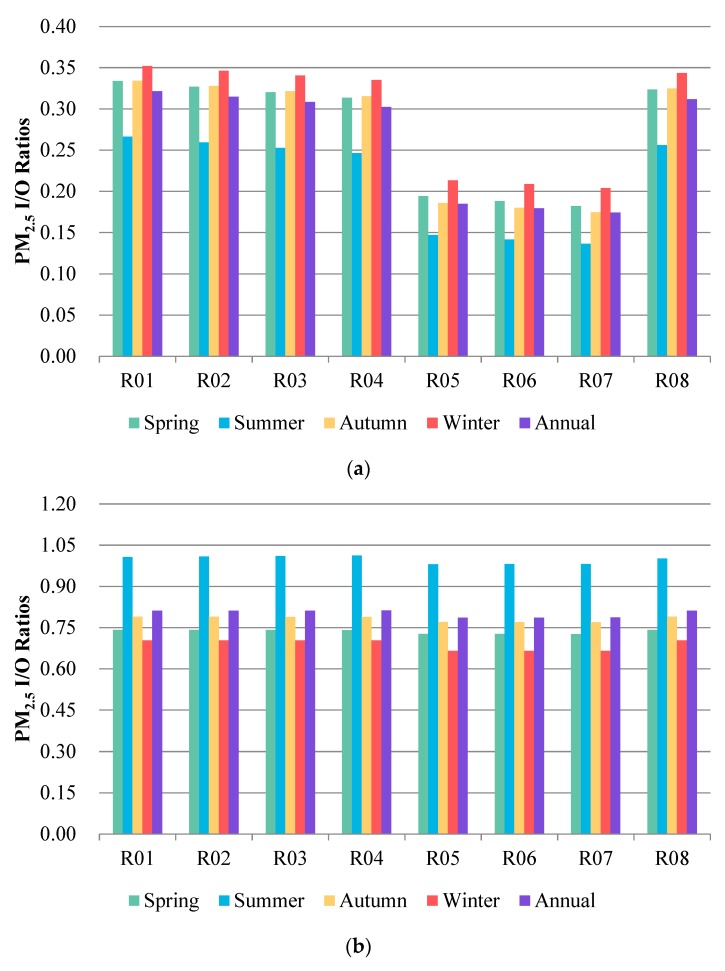
Average seasonal PM_2.5_ I/O ratios for different types of residences under Scenarios 1 (**a**) and Scenarios 2 (**b**).

The seasonally and annually averaged I/O ratios for different types of residences are shown in Figure 5. The simulation results show that high-rise residences often have relatively lower I/O ratios than multi-story buildings. The difference was more significant in scenario 1, with a nearly two-fold difference of the I/O ratio between multi-story and high-rise residences observed. In scenario 2, even though high-rise residences still showed lower I/O ratios than the multi-story buildings, the difference was much smaller compared to scenario 1. The windows and doors were closed in scenario 1 and the building characteristics may be the dominant influencing factor of outdoor PM_2.5_ infiltrations. According to the design standard for the energy efficiency of residential buildings in China [43], the airtightness of high-rise buildings should be higher than that of multi-story buildings. Therefore, the I/O ratios of high-rise buildings were much lower than those of multi-story residences. In scenario 2, window opening and cooking emissions were allowed, which increased indoor PM_2.5_ concentrations and reduced the difference in I/O ratios between different buildings. Building age is another influencing factor for outdoor PM_2.5_ infiltration especially in scenario 1. The older the building is, the higher I/O ratio it has. Similar conclusions can be found in previous studies, where older homes were often associated with a higher infiltration rate [19,27]_._ This can be attributed to changes in the building code and deterioration of the building over time [56].

From Figure 5, we can see considerable seasonal variability in the I/O ratios for different types of buildings, but the trends are quite different between the two scenarios. For scenario 1, the highest I/O ratios were found in the winter, and the lowest ratios occurred in the summer. Under scenario 1, outdoor PM_2.5_ was the only contributor to indoor PM_2.5_, and the I/O ratios varied synchronously with the outdoor PM_2.5_. The seasonal trends of I/O ratios were consistent with that of ambient PM_2.5_ [57]. In scenario 2, the trends were opposite of those in scenario 1. The highest I/O ratios were found in the summer, and the lowest ratios were found in the winter. The ventilation time in the summer (636 min/d) is over twice as much as that in the winter (281 min/d) (Table S2) [10]. The longer ventilation time can lead to an increase in the outdoor particle infiltration, which may be the possible reason for the higher I/O ratios in the summer than in the winter. The results are consistent with some other studies, which also found higher infiltration rates of particle matters in the summer than in the winter due to the frequency of opening windows in the summer [19,56]. In the colder winter period, occupants closed the windows of their residences to reduce heat loss. The reduction of ventilation time in the winter can reduce the outdoor air pollutants infiltration, but may also lead to the accumulation of air pollutants from indoor sources [58]. In this study, cooking emission is set as the only indoor source for PM_2.5_, and the release time of cooking lasted from half an hour to one hour every time. Kitchen fans were also set in the building models since the mechanical ventilation system is in accordance with the cooking schedule. The effect of mechanical ventilation and the relatively short period of cooking emission each day may reduce the influence of cooking emission on the average indoor PM_2.5_ concentration and the I/O ratios in the winter when the ventilation is insufficient. Therefore, the I/O ratios in the winter were lower than in the summer.

**Figure 6:**
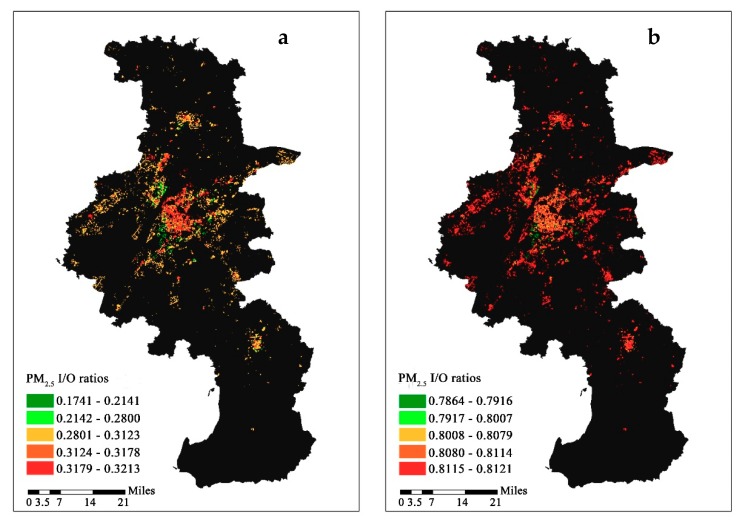
The annual PM_2.5_ I/O ratios for residences across Nanjing. (**a**): Scenarios 1, (**b**): Scenarios 2.

The spatial distribution of I/O ratios is shown in Figure 6. The spatial distributions were similar under the two scenarios. In scenario 1, residences with higher I/O ratios mainly existed in the old urban-core areas, including the Xuanwu, Qinhuai, and Gulou districts. This is likely due to multi-story and older residences being the dominant building type in the area. In the new urban areas, such as the Jianye, Yuhuatai, Jiangning, and Pukou districts, the number of high-rise residences was larger than that in the old urban-core area. Due to the protective effect of high-rise buildings, slightly decreased I/O ratios were observed around the old city center, especially in the area near the center. In suburban areas (i.e., Luhe, Lishui, and Gaochun), the I/O ratios were much higher than those in the new urban areas but lower than those in the center of the city. The spatial variation in I/O ratios across the city was smaller in scenario 2 than in scenario 1 due to the increase in indoor PM_2.5_ concentrations from the outdoor PM_2.5_ infiltration and indoor source emission. The I/O ratios in the old urban-core areas were also higher than those in the new urban areas, as shown in scenario 1. However, in the suburban areas, a slight increase in the I/O ratio was observed compared to that in the city center. In the suburbs of Nanjing, multi-story residential buildings were the dominant building type, and high-rise buildings were rarely distributed, which resulted in higher I/O ratios.

### 3.3. Spatial and Temporal Distributions of Indoor PM_2.5_ Concentrations

**Figure 7:**
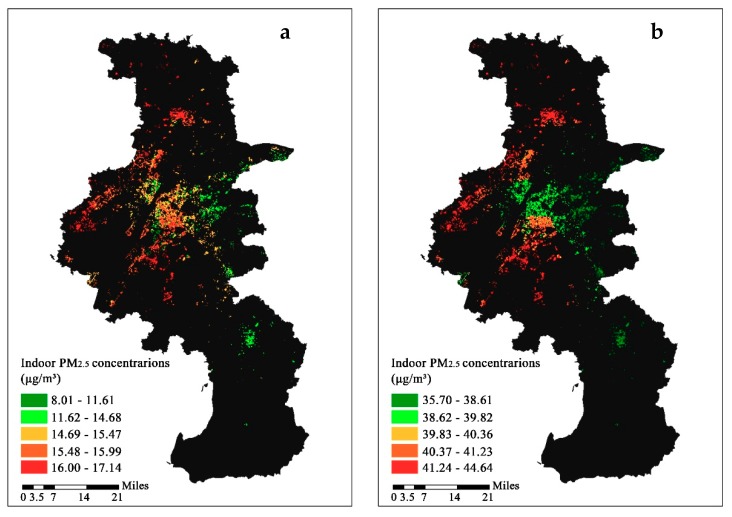
The spatial distributions of annual indoor PM_2.5_ concentrations estimated from outdoor PM_2.5_ concentrations. (**a**) Scenarios 1 and (**b**) Scenarios 2.

Indoor PM_2.5_ concentrations were calculated according to the outdoor concentrations and I/O ratios under the two scenarios. Figure 7 reveals a significant internal spatial difference in indoor PM_2.5_ levels across the city. The concentration ranges from 8.01 μg/m^3^ to 17.14 μg/m^3^, with a mean concentration of 14.75 ± 1.93 μg/m^3^ in scenario 1, while it ranges from 35.70 μg/m^3^ to 44.64 μg/m^3^, with a mean concentration of 39.87 ± 1.55 μg/m^3^ in scenario 2. The average concentration of indoor PM_2.5_ under the two scenarios were all lower than that of outdoors (49.28 μg/m^3^). The average concentration is higher under scenario 2, which is similar to the trend observed in the I/O ratios. When comparing with the indoor air quality guidelines for selected pollutants developed by the World Health Organization (WHO), the annual average indoor PM_2.5_ levels estimated in scenario 1 and 2 both exceeded the recommended value (10 μg/m^3^) [59,60].

The results of the indoor PM_2.5_ estimation are consistent with those reported in previous studies. The annual indoor PM_2.5_ concentration under scenario 2 (39.87 ± 1.55 μg/m^3^) is within the range of PM_2.5_ levels (37.08 μg/m^3^, 55.56 μg/m^3^, and 45.09 μg/m^3^ during the summer, winter, and transitional seasons, respectively) in residences of Nanjing measured by Shao et al. in 2016 [17]. The measurements of indoor PM_2.5_ concentrations in another study were much higher, with means of 40.40 μg/m^3^ and 64.90 μg/m^3^ in two dwellings [61]. Considering the variation of the source of indoor PM_2.5_ and influential factors [62], it is hard to compare the modeled concentrations with the measurements without considering indoor environmental conditions, such as building characteristics, occupant window-opening activities, indoor source emissions, and ventilation systems.

The seasonal variations of indoor PM_2.5_ in residences across Nanjing are shown in Figures S2 and S3. Considerable seasonal variability in indoor PM_2.5_ levels was observed under the two scenarios. In scenario 1, the highest indoor PM_2.5_ occurred in the winter, with a range of 23.54 ± 2.92 μg/m^3^, and the lowest occurred in the summer, with a range of 7.77 ± 1.09 μg/m^3^. Indoor PM_2.5_ levels during the transition seasons (i.e., spring and autumn) were 17.13 ± 2.23 μg/m^3^ and 11.42 ± 1.59 μg/m^3^, respectively. In scenario 2, the variation trends in indoor PM_2.5_ were similar to those in scenario 1, but the values were much higher under scenario 2. The highest indoor PM_2.5_ level was also observed in the winter, with a range of 49.81 ± 2.53 μg/m^3^, which was followed by the spring, autumn, and summer, with ranges of 40.78 ± 1.62 μg/m^3^, 31.86 ± 1.50 μg/m^3^, and 28.90 ± 1.29 μg/m^3^, respectively. The seasonal changes in indoor PM_2.5_ concentrations were consistent with those in the outdoor concentrations [57]. Similar change tendencies in indoor and outdoor PM_2.5_ concentrations indicated that outdoor-originating particles contributed the most to indoor PM_2.5_ concentrations in Nanjing.

Estimated indoor PM_2.5_ concentrations are a combination of I/O ratios and outdoor PM_2.5_ concentrations. Therefore, the spatial distribution of indoor PM_2.5_ levels (Figure 7) was influenced by the distributions of these two factors. Generally, the spatial distribution of indoor PM_2.5_ levels is consistent with that of the outdoors (Figures S4 and S5), which indicates that the spatial distribution of outdoor PM_2.5_ concentration was the dominant influence factors for PM_2.5_ concentrations in the residences. The spatial variation in indoor PM_2.5_ concentrations in scenario 2 was reduced compared to that in scenario 1 due to the decrease in spatial variation of I/O ratios. In the city center, in new urban district and southwest areas (where outdoor PM_2.5_ concentrations were higher), higher indoor PM_2.5_ concentrations were observed compared to those in the area to the southeast. However, the indoor PM_2.5_ concentrations in the center of the city or the new urban district were much lower than those in the suburban area even though the outdoor air pollution levels were similar. The lower estimated indoor PM_2.5_ concentrations in the urban area was due to the attenuation of the predominant building type (high-rise and newly built buildings) and their lower I/O ratios. The results indicate that building type plays a significant role in the spatial distribution of indoor PM_2.5_ levels, which should be considered when exploring the internal differences in indoor air pollutants within a city.

### 3.4. Exposure Estimation and Health Impact Evaluation

Based on the average indoor PM_2.5_ concentrations estimated in this study, outdoor PM_2.5_ concentrations, and the time residents spent indoors and outdoors, human exposure to PM_2.5_ concentrations in Nanjing was estimated. The PM_2.5_ exposure and health impact assessment were mainly calculated under scenario 2, which represents the concentration level of PM_2.5_ in residences under the normal living conditions. Based on the estimated indoor PM_2.5_ concentration in scenario 2, outdoor PM_2.5_ concentrations and time spent indoors and outdoors, the annual mean PM_2.5_ exposure concentration in Nanjing was estimated to be 41.06 μg/m^3^, which is lower than the outdoors (49.28 μg/m^3^). If the average outdoor PM_2.5_ concentration is used to substitute people’s exposure concentration to PM_2.5_, it will lead to a 16.67% overestimation.

The RRs for the four diseases were 1.33, 1.59, 1.25, and 1.58 for IHD, stroke, LC, and COPD, respectively. Based on the calculated RRs for the four diseases and the population data of Nanjing, the number of premature deaths attributable to PM_2.5_ exposure in 2016 were estimated to be 2498, 3866, 687, and 802 for IHD, stroke, LC, and COPD, respectively. If the PM_2.5_ exposure concentrations were simply substituted with the ambient concentrations, the results would be 6.44%, 8.74%, 11.80%, and 11.53% overestimated for IHD, stroke, LC, and COPD, respectively. Indoor air exposure needs to be taken into account to improve the accuracy of exposure and health impact assessments in epidemiological studies.

There are a number of limitations that need to be considered in this study. First, due to the lack of information on the building form, the buildings in Nanjing were divided into multi-story and high-rise buildings and were further classified by their construction year. This combination of the building story and age is rough, and detailed information on these buildings, such as the building form, would be needed for a more precise analysis in the future. Second, the residential building data of Nanjing were insufficient. The lack of information on building age for a portion of the residences (R08) may lead to some uncertainties in indoor PM_2.5_ estimation. Scenario 1 was chosen as an example for the uncertainty analysis since the indoor PM_2.5_ concentrations were more sensitive to the age of the buildings under this scenario. The average indoor PM_2.5_ concentrations were 14.55 μg/m^3^ and 15.79 μg/m^3^, respectively, when the I/O ratios of the oldest and newest multi-story buildings were used to represent the I/O ratio of building R08. The difference was small when compared to the value calculated from the mean I/O ratio of multi-story buildings (14.75 μg/m^3^), which indicates that the lack of information on building age for some residences had less influence on the estimation of indoor PM_2.5_ levels in the city. Third, in the early 1990s, some high-rise residences were built as tower buildings. However, due to the lack of information on building form in the building database, the distribution of this type of building was unknown. The I/O ratio for high-rise tower buildings built in the period from 1991 to 2000 was modeled to predict indoor PM_2.5_ concentrations in the city. Since the number of this type of building was very small (0.33%), the influence was found to be very small. The average indoor PM_2.5_ concentration estimated from the high-rise tower buildings (14.76 μg/m^3^) was approximately equal to that of the slab-type high-rise buildings (14.75 μg/m^3^).

## 4. Conclusions

This study modeled the spatiotemporal variations of PM_2.5_ concentrations in residences in Nanjing with different building characteristics using the CONTAM model. The ability of the CONTAM model to predict indoor PM_2.5_ in multiple residences was validated in this study, which would support the application of the model for a broad range of indoor PM_2.5_ predictions in Chinese residences in the future. The spatial and temporal variations of I/O ratios and indoor PM_2.5_ levels in Nanjing were studied based on the model results and building distribution data. Indoor PM_2.5_ concentrations were observed to be lower than outdoors especially when the windows were closed due to the protection of the building envelope. Seasonal variations of PM_2.5_ in residences were observed in this study, with the highest concentrations occurring in the winter and the lowest occurring in the summer, which is consistent with the variation trends of outdoor PM_2.5_. The results indicated that the major source of indoor PM_2.5_ pollution is the penetration of the particles from the ambient environment. During heavy-pollution days (e.g., in winter), people can reduce the time of outdoor activities and indoor ventilation when there is no significant indoor source, to reduce their exposure to outdoor air pollutions.

Building characteristics play an important role in the distribution of PM_2.5_ levels in residences. Older multi-story residential buildings were found to be more easily affected by outdoor PM_2.5_ pollutions than newly built high-rise buildings, which indicated that occupants living in older multi-story residences may be more sensitive and need more protection from ambient air pollution. The spatial distribution of indoor PM_2.5_ over Nanjing were observed in this study. Higher indoor PM_2.5_ concentrations were observed in the city center, new urban district, and the southwest area of the city due to the effect of building characteristics on outdoor PM_2.5_ permeability. The results indicate that building type plays a significant role in the spatial distribution of indoor PM_2.5_ levels, which should be considered when exploring the internal differences in indoor air pollutants within a city. The overall population exposure to PM_2.5_ and the health consequences in Nanjing would be overestimated if the indoor air concentrations were simply substituted with the outdoor values. This demonstrates that efforts to reduce human exposure to PM_2.5_ and its health impacts need to be applied based on both outdoor PM_2.5_ concentrations and building characteristics, which could influence the indoor PM_2.5_ concentrations within a city.

## Figures and Tables

**Table 1:** Residential building descriptions and building input parameters of the model.

Building Code	Construction Year	Building Story	Permeability of Exterior Door and Windows ^a^ (m^3^/(m^2^·h))	Effective Leakage Area of the Exterior Wall ^b^ (cm^2^/m^2^)
R01	Before 1990	Multistory	7.5	1.88
R02	1991–2000	Multistory		1.69
R03	2001–2010	Multistory		1.52
R04	2011–now	Multistory		1.36
R05	1991–2000	High-rise	4.5	1.08
R06	2001–2010	High-rise		0.97
R07	2011–now	High-rise		0.87
R08 ^c^	Unknown	Multistory	7.5	-

^a^ The permeability of exterior doors and windows for each building was obtained from the design standard for the air permeability performances of multistory and high-rise residences [42]. ^b^ The effective leakage areas of the exterior were estimated according to the empirical model proposed by Chan et al. based on the floor area and construction year [45]. ^c^ For building code R08, the construction year was unknown, and the effective leakage area of the exterior wall cannot be estimated by the empirical model [45].

## Supplementary Materials

The following are available online at http://www.mdpi.com/1660-4601/16/1/144/s1, Table S1: PM_2.5_ emission rate, penetration factor, and deposition rate. Table S2: Schedule of ventilation used as inputs for CONTAM simulation. Table S3: Fit parameters of four diseases for IER model. Table S4: Air leakage area for different residences in the CONTAM model. Table S5: Variations in parameter inputs for the sensitivity analysis. Table S6: Variations in the average annual I/O ratio for different parameters. Figure S1: The layout of multi-story and high-rise residential buildings. Figure S2: Seasonally averaged indoor PM_2.5_ concentrations in residences in Nanjing (Scenario 1). Figure S3: Seasonally averaged indoor PM_2.5_ concentrations in residences in Nanjing (Scenario 2). Figure S4: The spatial distribution of indoor/outdoor PM_2.5_ concentrations in residences across Nanjing (scenario 1). Figure S5. The spatial distribution of indoor/outdoor PM_2.5_ concentrations in residences across Nanjing (scenario 2).
