# Supplementary material for: Spatiotemporal Variations of Indoor PM2.5 Concentrations in Nanjing, China
Source: Int J Environ Res Public Health. 2019 Jan 7;16(1):144. doi: 10.3390/ijerph16010144 (PMC6339030; doi:10.3390/ijerph16010144)
Supplement: Supplementary file 1 [file ijerph-16-00144-s001.pdf]

International Journal of
Environmental Research and Public Health

MDPI

Supplementary

Spatiotemporal variations of indoor $PM_{2.5}$ concentrations in Nanjing, China

Zhijuan Shao [1], Xiangjun Yin [2], Jun Bi [1,3], Zongwei Ma [1,3*], Jinnan Wang [1,4]

[1] State Key Laboratory of Pollution Control & Resource Reuse, School of the Environment, Nanjing University, Nanjing, China
[2] Nanjing Urban Planning & Research Center, Nanjing, China
[3] Jiangsu Collaborative Innovation Center of Atmospheric Environment and Equipment Technology, Nanjing University of Information Science & Technology, Nanjing, China
[4] State Environmental Protection Key Laboratory of Environmental Planning and Policy Simulation, Chinese Academy for Environmental Planning, Beijing, China
* Correspondence: zma@nju.edu.cn; Tel.: +86-25-89681526

Contents

CONTAM building model
Sensitivity analysis
References

Tables

Table S1 $PM_{2.5}$ emission rate, penetration factor, and deposition rate.
Table S2 Schedule of ventilation used as inputs for CONTAM simulation.
Table S3 Fit parameters of four diseases for IER model.
Table S4 Air leakage area for different residences in the CONTAM model.
Table S5 Variations in parameter inputs for the sensitivity analysis.
Table S6 Variations in the average annual I/O ratio for different parameters.

Figures

Figure S1. The layout of multistory and high-rise residential buildings.
Figure S2. Seasonally averaged indoor $PM_{2.5}$ concentrations in residences in Nanjing (Scenario 1).
Figure S3. Seasonally averaged indoor $PM_{2.5}$ concentrations in residences in Nanjing (Scenario 2).
Figure S4. The spatial distribution of indoor/outdoor $PM_{2.5}$ concentrations in residences across Nanjing (scenario 1).
Figure S5. The spatial distribution of indoor/outdoor $PM_{2.5}$ concentrations in residences across Nanjing (scenario 2).

Table 1. $PM_{2.5}$ emission rate, penetration factor, and deposition rate.

Source	Penetration Factor	Emission Rate	Deposition Rate
Outdoor infiltration	0.8; closed windows	/	0.19 /h [1]
	1.0; open windows	/	
Cooking emission	/	1.56 mg/min [2]	0.19 /h [1]

Table S2. Schedule of ventilation used as inputs for CONTAM simulation.

Activity	Season	Schedule	Ventilation Time (min/d)
Window opening	Spring	7:00–9:00; 11:00–15:00; 17:00–18:34;	454
	Summer	0:00–7:36; 21:00–24:00	636
	Autumn	7:00–9:00; 11:00–15:00; 17:00–18:34;	454
	Winter	7:00–8:00; 11:00–13:00; 17:00–18:41	281

Table S3. Fit parameters of four diseases for IER model [3].

	IHD	stroke	COPD	LC
α	0.843	1.01	18.3	159
γ	0.0724	0.0164	0.000932	0.000119
δ	0.544	1.14	0.682	0.735
C_0	6.96	8.38	7.17	7.24

1. CONTAM building models

1.1. The layout of residential buildings

The multistory residential buildings were modeled as slab-type buildings, as described by Shi et al.,[4] with two apartment units sharing one floor and one cell consisting of one floor and adjacent apartment units. High-rise residential buildings were modeled as slab-type buildings, with four apartment units sharing a corridor and two elevators. In the 1990s, some high-rise residential buildings were built as tower buildings; therefore, the high-rise tower buildings built in this period were also considered and discussed in this study. A family with three people was simulated to live in each apartment unit, with an area of 109.5 m^2 calculated via the per capita floor space (36.5 m^2/ per capita) of the urban residents in Nanjing. Fig.S1. shows the layout of the multistory and high-rise residential buildings.

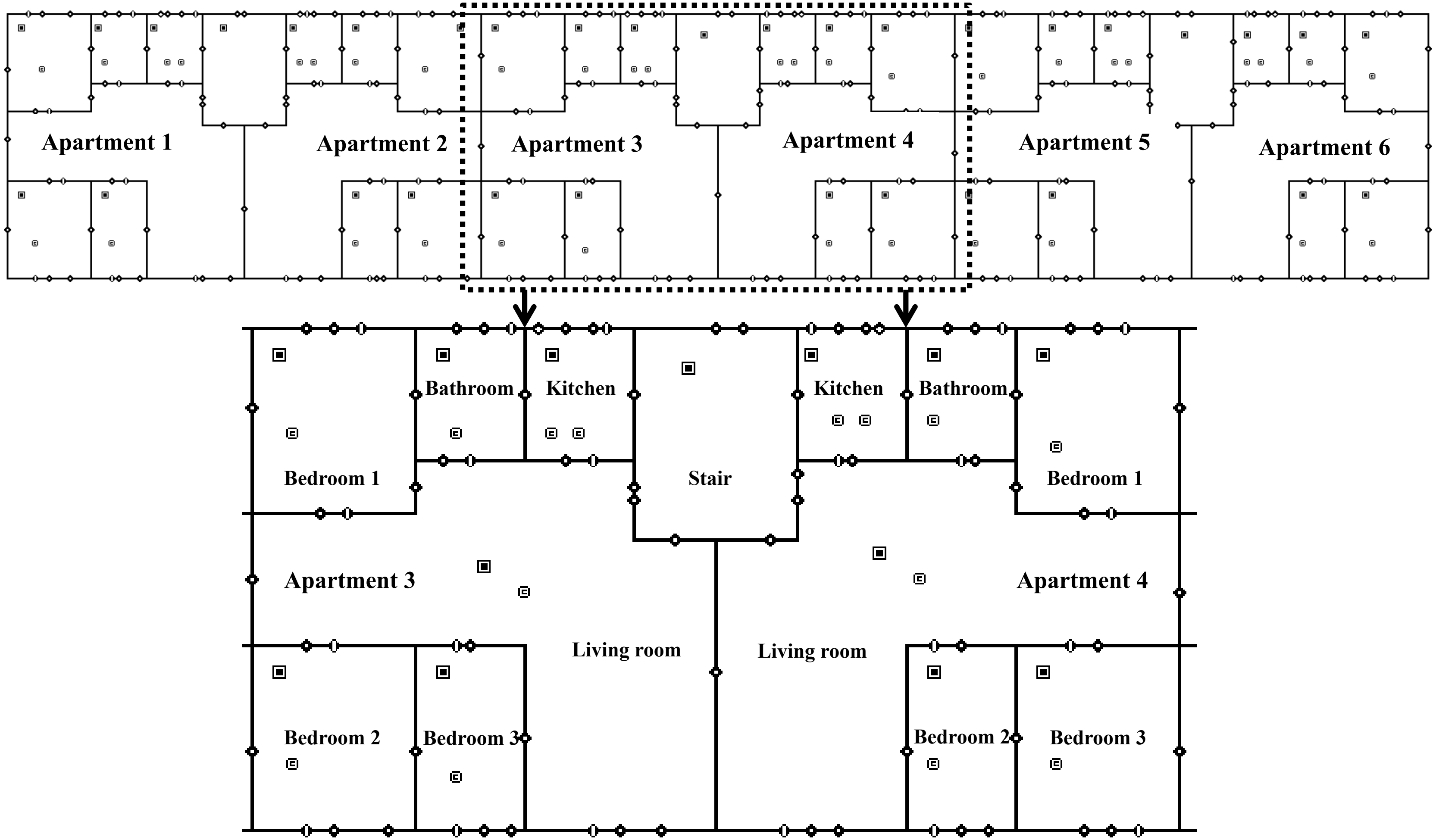

(**a**) Multistory buildings

Figure S1. The layout of multi-story and high-rise residential buildings.

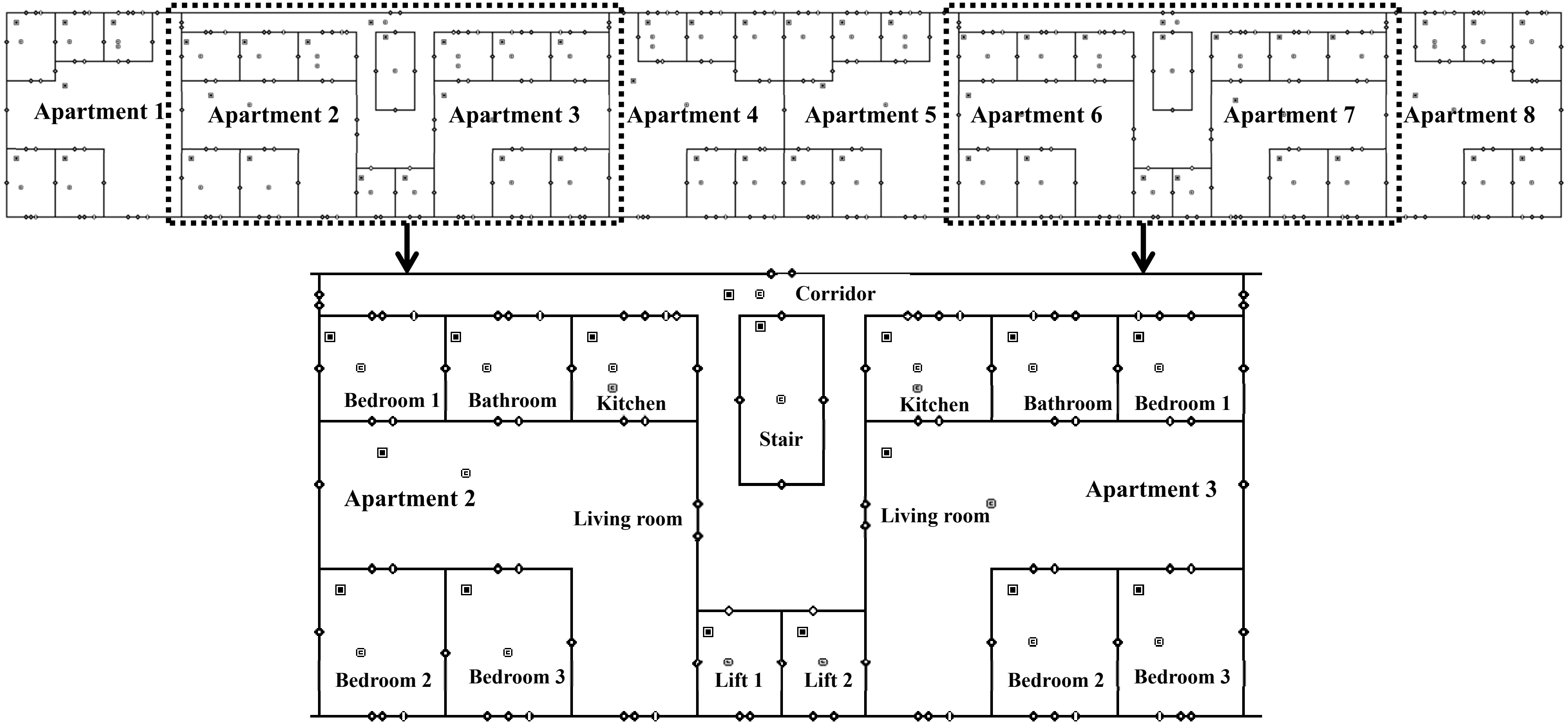

(**b**) High-rise building (slab-type)

Figure S1. (Continued) The layout of multi-story and high-rise residential buildings.

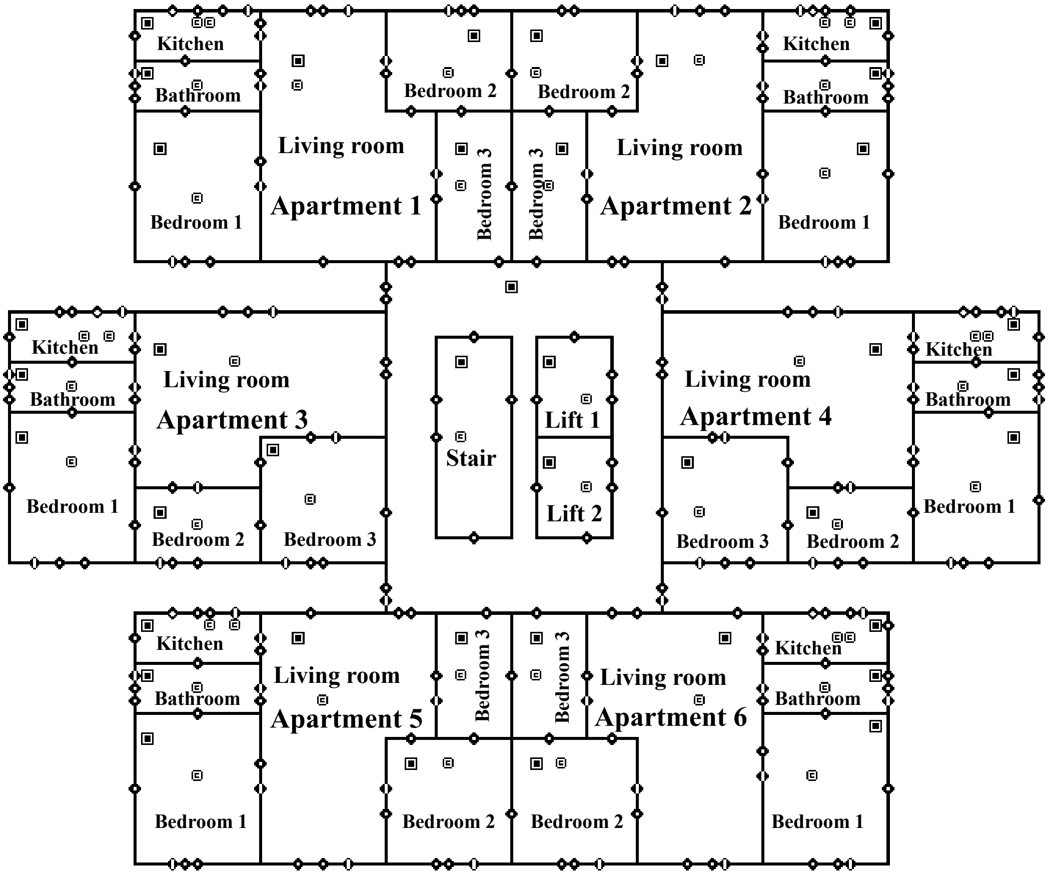

(**c**) high-rise building (Tower-type)

Figure S1. (Continued) The layout of multistory and high-rise residential buildings.

1.2. Airflow paths of building models

The airflow paths considered for each buildings include the leakage area of exterior walls, doors, windows, interior door, stairwell, elevator shaft. The exterior wall leakage was modeled using an effective leakage area calculated based on the empirical model proposed by Chan et al. [5] using the floor area and construction year. Although the empirical model was mainly constructed for low-rise single-family dwellings [5], apartment buildings can be considered as one-floor buildings, with the same construction year and floor area per floor, to extrapolate the empirical model for high-rise buildings, as suggested by Shi et al [4]. Closed windows and door leakages in the building were modeled using the effective leakage area, which was calculated according to the design standard for the air permeability performances of multistory and high-rise residences in hot summer and cold winter zones [6,7]. Other airflow path elements, including internal walls, floors, ceilings, stairwell, and elevator shaft were modeled according to the input data library for the multizone airflow and indoor air quality analysis. The corresponding best estimate values from the library were selected and used in this study [8]. A two-way flow model was assumed when the windows were open. The penetration rate of outdoor $PM_{2.5}$ was set according to the status of the windows (closed/opened), as shown in Table S1. Table S4 provides details of the CONTAM parameters used in the model.

Table S4. Air leakage area for different residences in the CONTAM model.

Building Component	Effective Leakage Area
Exterior door (closed)*	
Multistory residence	17.07 cm^2 /unit
High-rise residence	10.24 cm^2/unit
Exterior windows (closed)*	
Multistory residence	15.36 cm^2 /unit
High-rise residence	9.21 cm^2/unit
Interior walls	2.00 cm^2/m^2
Floor	2.20 cm^2/m^2
Ceiling	1.80 cm^2/m^2

* The effective leakage areas for exterior doors and windows were calculated according to the equation [9]: $Q = C_d A\sqrt{2\Delta P/\rho}$, where Q—volume airflow (m^3/s), Cd—discharge coefficient, A—the leakage area (m^2), and ϱ—air density (kg/m^3)

2. Sensitivity analysis

To explore the sensitivity of CONTAM model to variations in the input parameters, a sensitivity analysis was carried out in this study. The sensitivity analysis was conducted under scenarios 1 and 2. The R03 and R06 building models were used for the sensitivity analysis as representatives of multistory buildings and high-rise buildings, respectively. The exterior wall leakage area, leakage area of exterior doors/windows, $PM_{2.5}$ penetration factor, deposition rate and cooking emission rate were used for the sensitivity analysis. Parameter variations were selected based on the range of particle behaviors and building characteristics available in the literature and the building design standard for different type of residences (Table S5). The maximum and minimum alternative values of each input parameter were entered into the model for sensitivity analysis while holding all other variables constant. The percent differences in $PM_{2.5}$ I/O ratios from the baseline were calculated to demonstrate the model sensitivity to each input variable.

The results of the sensitivity analysis for scenarios 1 and 2 can be seen in Table S6. The results indicate a high levels of model sensitivity to the $PM_{2.5}$ penetration factor and deposition rate. The penetration factor and deposition rate are two key parameters that impact indoor-outdoor particle dynamics. The sensitivity of the CONTAM model to the two parameters has also been demonstrated in previous studies [10, 11]. The sensitivity of the predicted I/O ratios also varied with the building type. High-rise residences showed more sensitivity to variations in the model inputs compared to multistory buildings. In scenario 1, the leakage area of the exterior doors/windows and the particle penetration factor had large influences on the I/O ratio, while in scenario 2, the influences of the two parameters were much smaller. The difference between the two scenarios was easy to understand. In scenario 1, all of the windows were closed, and outdoor air infiltration was the only source for indoor $PM_{2.5}$ pollution; therefore, the model was sensitive to the two key parameters, which impacted indoor-outdoor particle dynamics and the penetrability of the building. In scenario 2, when the windows were opened and indoor cooking source existed, the sensitivity of the model to exterior doors/windows and the penetration factor was reduced due to the larger influences of the cooking emission rate and deposition rate.

Table S5. Variations in parameter inputs for the sensitivity analysis.

Parameter	Category	Variation	Reference
Effective leakage area of exterior walls (cm^2/m^2)	Ex1	1.59 (R03)<br>1.01 (R06)	[5]
	Ex2	1.44 (R03)<br>0.92 (R06)	
Effective leakage area of exterior doors/windows (cm^2/unit)	Win1	Doors: 17.07 (multistory building)<br>10.24 (high-rise building)<br>Windows: 15.36 (multistory building)<br>12.29 (high-rise building)	[6,7]
	Win2	Doors: 13.66 (multistory building)<br>6.83 (high-rise building)<br>Windows: 12.29 (multistory building)<br>6.15 (high-rise building)	
Penetration factor	P1	0.6	[12]
	P2	1.0	
Deposition rate (/h)	D1	0.06	[13]
	D2	0.39	
Emission rate (mg/min)	E1	0.02	[14]
	E2	10.02	

Table S6. Variations in the average annual I/O ratio for different parameters

Parameter		Variations (scenario 1)		Variations (scenario 2)	
		Multistory	High-rise	Multistory	High-rise
Effective leakage area of exterior walls	Ex1	0.85%	1.13%	0.02%	0.00%
	Ex2	1.00%	1.45%	0.02%	0.00%
Effective leakage area of exterior doors/windows	Win1	/	/	/	/
	Win2	6.09%	8.61%	0.34%	0.00%
Penetration factor	P1	11.31%	25.11%	2.20%	3.01%
	P2	11.31%	25.03%	1.36%	3.01%
Deposition rate	D1	93.44%	132.18%	21.59%	26.27%
	D2	41.17%	47.14%	17.15%	20.38%
Emission rate	E1	/	/	10.61%	12.78%
	E2	/	/	58.36%	70.27%

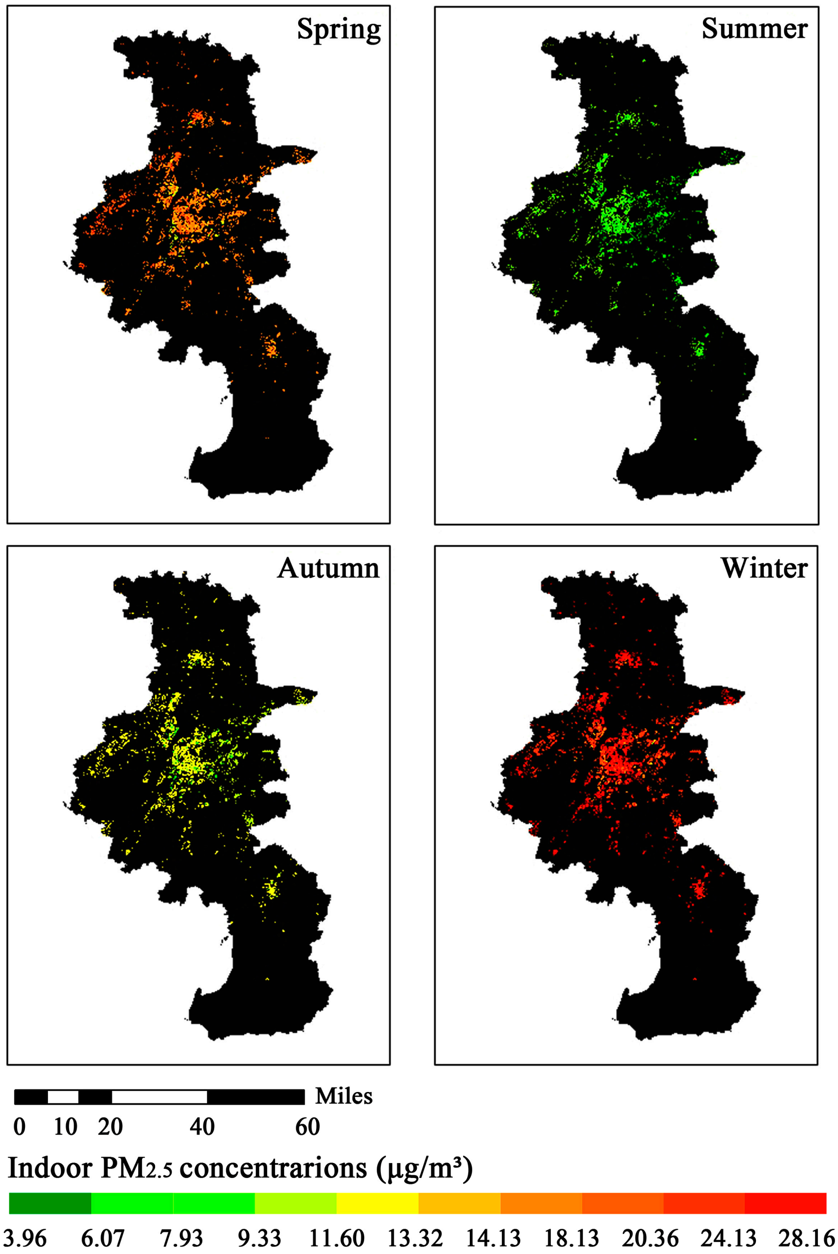

Figure S2. Seasonally averaged indoor $PM_{2.5}$ concentrations in residences in Nanjing (Scenario 1).

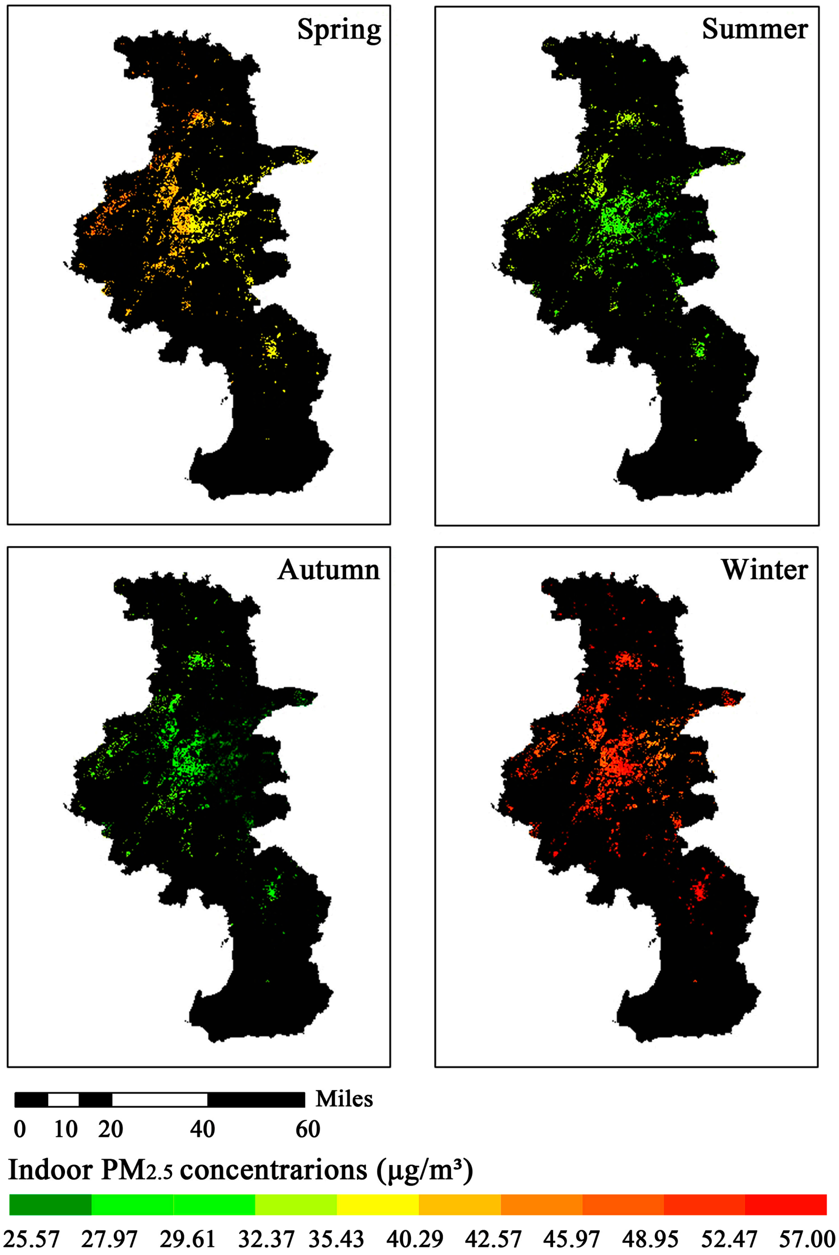

Figure S3. Seasonally averaged indoor $PM_{2.5}$ concentrations in residences in Nanjing (Scenario 2).

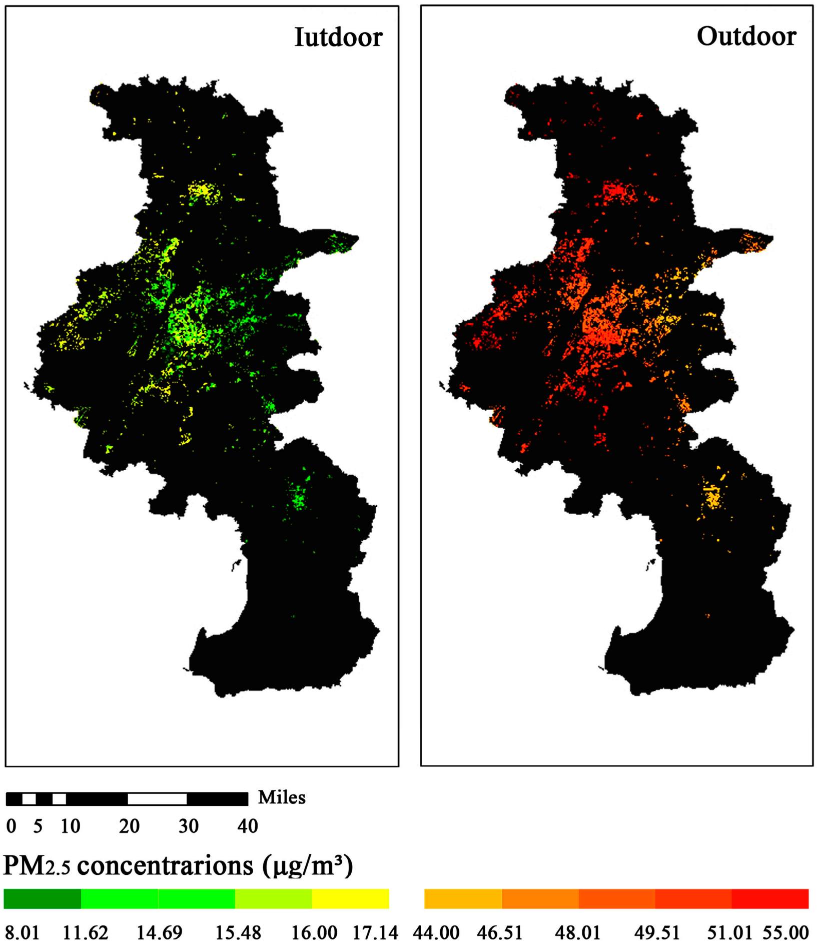

Figure S4. The spatial distributions of indoor/outdoor $PM_{2.5}$ concentrations in residences across Nanjing (scenario 1).

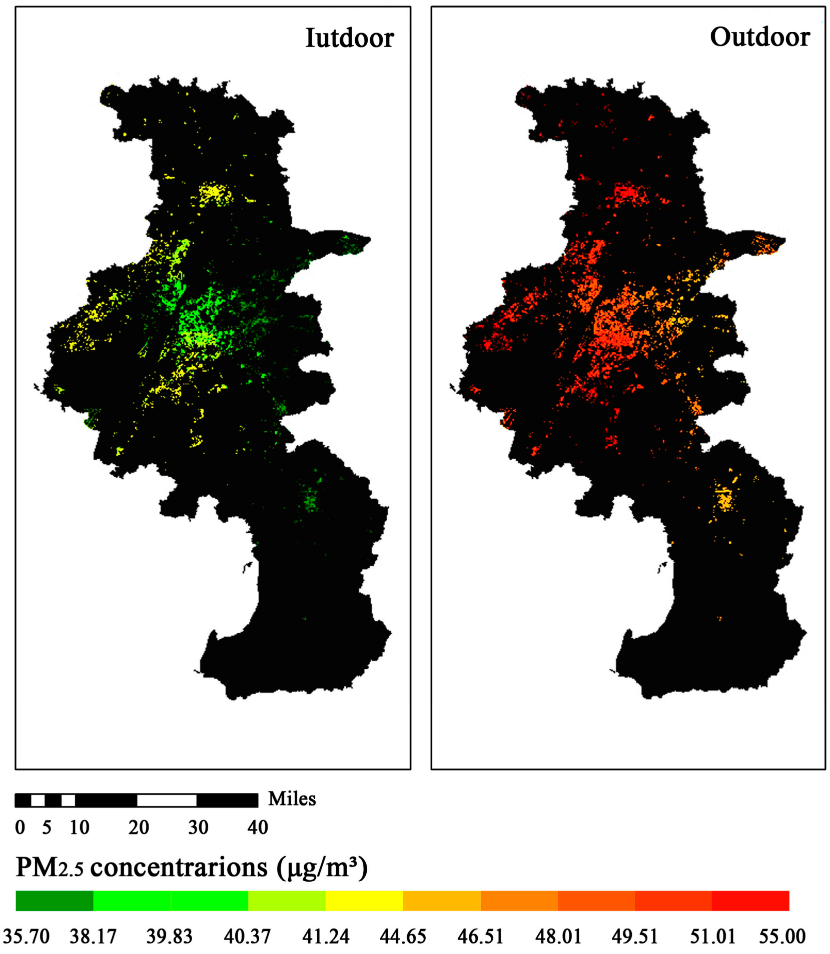

Figure S5. The spatial distributions of indoor/outdoor $PM_{2.5}$ concentrations in residences across Nanjing (scenario 2)

References

1. Long, C.M.; Suh, H.H.; Catalano, P.J.. Koutrakis, P., Using time- and size-resolved particulate data to quantify indoor penetration and deposition behavior. *Environ. Sci. Technol*. **2001**, *35*, 2089-2099.
2. Burke, J.M.; Zufall, M.J.. Ozkaynak, H., A population exposure model for particulate matter: case study results for $PM_{2.5}$ in Philadelphia, PA. *J. Expo. Anal. Environ. Epidemiol*. **2001**, *11*, 470-489.
3. Jiang, X.; Zhang, Q.; Zhao, H.; Geng, G.; Peng, L.; Guan, D.; Kan, H.; Huo, H.; Lin, J.; Brauer, M.; Martin, R.V.. He, K., Revealing the Hidden Health Costs Embodied in Chinese Exports. *Environ. Sci. Technol*. **2015**, *49*, 4381-4388.
4. Shi, S.; Chen, C.. Zhao, B., Air infiltration rate distributions of residences in Beijing. *Build. Environ*. **2015**, *92*, 528-537.
5. Chan, W.Y.R.; Nazaroff, W.W.; Price, P.N.; Sohn, M.D.; Gadgil, A.J., Analyzing a database of residential air leakage in the United States. *Atmos. Environ*. **2005**, *39*, 3445-3455.
6. General Administration of Quality Supervision, Inspection and Quarantine of the People's Republic of China. *Graduations and Test Methods of Air Permeability, Watertightness, Wind Load Resistance Performance for Building External Windows and Doors, GB/T 7106-2008*; 2008.
7. Ministry of Housing and Urban-Rural Development of the People's Republic of China. *Design Standard for Energy Efficiency of Residential Buildings in Hot Summer and Cold Winter Zone*. Industry standards of the People's Republic of China JGJ 134-2010, 2010.
8. Persily, A.K.; Ivy, E.M. *Input Data for Multizone Airflow and IAQ Analysis, NISTIR 6585 2001*, National Institute of Standards and Technology.
9. Hazim B, A.; Li, X.; Zhao, B.; Shao, X.. Cai, H., Ventilation of buildings, China Machine Press, Beijing, China, 2011.
10. Rim, D.; Persily, A.; Emmerich, S.; Dols, W.S.. Wallace, L., Multi-zone modeling of size-resolved outdoor ultrafine particle entry into a test house, *Atmos. Environ*. **2013**, *69*, 219–230.
11. Shrubsole, C.; Ridley, I.; Biddulph, P.; Milner, J.; Vardoulakis, S.; Ucci, M.; Wilkinson, P.; Chalabi, Z.. Davies, M., Indoor $PM_{2.5}$ exposure in London's domestic stock: Modelling current and future exposures following energy efficient refurbishment, *Atmos. Environ*. **2012**, *62*, 336–343.
12. Chen, C.; Zhao, B., Review of relationship between indoor and outdoor particles: I/O ratio, infiltration factor and penetration factor, *Atmos. Environ*. **2011**, *45*, 275–288.
13. Ozkaynak, H.; Xue, J.; Spengler, J.; Wallace, L.; Pellizzari, E.. Jenkins, P., Personal exposure to airborne particles and metals: Results from the particle team study in Riverside, California, *J. Expo. Anal. Environ. Epidemiol*. **1996**, *6*, 57–78.
14. Chen, C.; Zhao, Y.; Zhao, B., Emission Rates of Multiple Air Pollutants Generated from Chinese Residential Cooking, *Environ. Sci. Technol*. **2018**, *52*, 1081–1087.
